# The role of electrical stimulation in the management of avascular necrosis of the femoral head in adults: a systematic review

**DOI:** 10.1186/s12891-017-1663-5

**Published:** 2017-07-28

**Authors:** Talal Al-Jabri, Jessica Yan Qi Tan, Gabriel Yihan Tong, Ravikiran Shenoy, Babar Kayani, Timothy Parratt, Tahir Khan

**Affiliations:** 1 Royal National Orthopaedic Hospital, Brockley Hill, Stanmore, Middlesex, HA7 4LP UK; 2 Queen Mary University of London, Whitechapel Rd, London, E1 1BB UK; 3 Great Ormond Street Hospital, Great Ormond St, London, WC1N 3JH UK

**Keywords:** Avascular necrosis, Osteonecrosis, Hip, Femoral head, Electrical stimulation

## Abstract

**Background:**

Avascular necrosis of the femoral head causes significant morbidity and occurs in up to 20,000 people per year. A variety of nonoperative and operative measures have been trialled however a definitive treatment algorithm is yet to be established. Young adults in many cases have undergone multiple surgical procedures in their lifetime with increasing risks of complications. Less invasive techniques may help reduce the number of operations required and positively influence the natural history of the disease process. Our aim was to navigate the literature and examine the results of electrical stimulation of the femoral head in avascular necrosis.

**Methods:**

The following defined search strategy was used to perform a systematic review using MEDLINE and Google Scholar databases: ((avascular necrosis) OR (osteonecrosis)) AND (femoral head) AND ((electrical stimulation) OR (capacitive coupling) OR (pulsed electromagnetic fields)). Articles were reviewed and data compiled into tables for analysis.

**Results:**

Fourty six articles were identified with a total of 10 articles meeting the inclusion criteria. 8 articles were prospective studies and 2 were retrospective. Early Ficat stages showed the best responses to treatment via pulsed electromagnetic fields with improvements in both clinical and radiographic parameters. Direct current and capacitative coupling have had a more ambiguous outcome.

**Conclusions:**

Pulsed electromagnetic fields may have a role in the management of early avascular necrosis. The paucity of clinical studies into this technique indicates a need for further studies.

## Background

Avascular necrosis (AVN) of the femoral head is a debilitating, progressive condition which occurs in up to 20,000 people in the United States per year [1–3]. It can occur at any age however, typically adults in their third and fourth decades are affected. It frequently results in subchondral collapse and secondary osteoarthritis as the disease process progresses limiting the treatment options available and ultimately, necessitating a total hip arthroplasty. The pathophysiology has not been clearly defined however various mechanisms have been implicated and specific risk factors have been associated with the development of AVN. These include smoking, corticosteroid administration, diabetes mellitus, systemic lupus erythematous, rheumatoid arthritis and sickle cell disease amongst others [3–6].

Both nonsurgical and surgical treatment options have been used with varying rates of success nonetheless a specific algorithm for the various options has not yet been established. Importantly, young adult patients would in many cases require more than one arthroplasty procedure in their lifetime [7, 8] and as such interest in less invasive techniques aimed at slowing or preventing disease progression have gained the interest of clinicians involved in the management of AVN.

Electrical fields in bone known as strain related potentials arise from mechanical deformation of bone. These strain related potentials transfer information to the osteocyte regarding it’s biophysical environment. The use of exogenous electrical currents of the correct amplitude and frequency have been shown to have positive effects on bone formation, bone graft incorporation and bone repair in in vivo and in vitro models [6, 9]. Pulsed electromagnetic fields have been shown to decrease parathyroid hormone receptor activity on osteoblasts and to reduce the lysosomal content of osteoclasts thereby suppressing bone resorption and increasing bone mass [9].

Noninvasive techniques of applying electric fields include inductive or capacitive coupling. Capacitive coupling involves centring skin electrodes posteriorly and anteriorly to the femoral head. Inductive coupling involves pulsed, time-varied electromagnetic fields created by an external generator and a current carrying coil. Invasive techniques whereby an implantable current generating unit supplies a constant direct current (DC) have been described in the literature and often these involve implanting the cathode to the site of bone repair and the anode in the nearby soft tissues. This is usually done in conjunction with a core decompression and necessitates surgical removal following treatment is accomplished [6, 10, 11].

## Aims and objectives

The aim of this systematic review is to examine the published clinical and radiographic outcomes following the use of electrical stimulation in the management of avascular necrosis of the femoral head in adults.

## Methods

This systematic review was completed in accordance with the Preferred Reporting Items for Systematic Reviews and Meta-Analyses (PRISMA) reporting guidelines for the meta-analysis of intervention trials [12]. The protocol was not registered and ethical approval was not required as this was a small study involving review of existing, published literature and did not involve the handling of new patient data.

The following search strategy was used to complete a search on MEDLINE and Google Scholar from 1928 to April 2016: ((avascular necrosis) OR (osteonecrosis)) AND (femoral head) AND ((electrical stimulation) OR (capacitive coupling) OR (pulsed electromagnetic fields)). Journals in all languages were included, and there were no limitations on the search strategy. Abstracts were screened and articles relevant to the role of electrical stimulation for avascular necrosis were selected and included. Exclusion criteria included studies which did not separate Perthe’s disease from avascular necrosis of the femoral head in adults. Letters, editorials and review articles were excluded.

The technique of electrical stimulation used, duration of treatment, staging of avascular necrosis, follow-up period and complication rates were extracted from each article and compiled into a database. References of selected full text articles were screened for the inclusion of additional articles. Recorded data was extracted and entered into an excel spreadsheet (Microsoft Office Excel, 2007). The references were independently reviewed by 2 of the authors and any ambiguity was resolved through discussion. Bias was assessed and its influence if any included within the analysis as laid out by the Critical Appraisal Skills Programme [13]. Outcome measures have been summarized alongside individual studies in this systematic review as studies on this topic are limited in number, size, quality of research methodology and there is heterogeneity in the methodology used.

## Results

**Fig. 1:**
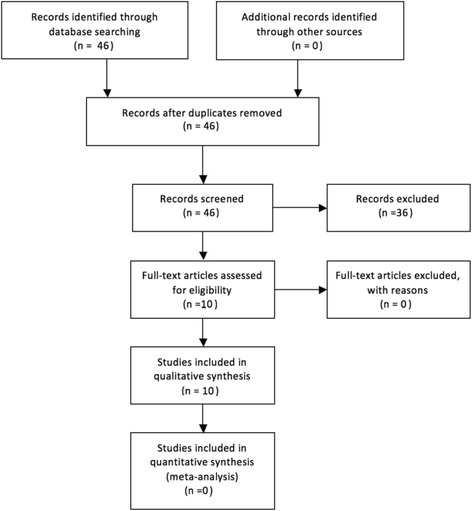
Flow chart presenting articles for inclusion in review

The role of electrical stimulation in femoral heads with avascular necrosis is a subject that has not been widely investigated. Of the 46 articles identified in our search, 36 did not meet our inclusion criteria or were duplicates, letters, editorials or review articles. Of the 10 papers included, 2 were retrospective studies, 8 were prospective studies (Table 1, Fig.1).

**Table 1:** Summary of results

Reference	Study type	Number of patients	Stage	Pre-treatment hip outcome score	Aetiology	Clinical technique	Additional management	Results	Post-treatment hip outcome score	Other
J.L. Cebrián et al. [3].	Retrospective	51 (70 hips)	ARCO staging used. I: 20 II: 50	Symptomatic, AVN + no collapse on MRI and X-ray	Idiopathic: 40 Steroids: 26 Alcohol: 4	1 pair of coils attached anteriorly & posteriorly, held in place over greater trochanter on molded splint. Single pulse of Frequency: 75 Hz Intensity: 400 mA Time: 1 at 3 ms Duration of tx: Coils worn for 8 h/day for 6 months	-	Follow up at 3, 6, 12, 24, 48 months with AP + Axial X-ray and MRI. Mean follow-up = 26 months. **Collapse** ARCO I + IIA: 0 collapse ARCO IIB: 3 collapse ARCO IIC: 5 collapse **D’Aubigne pain scale** Improve: 55 Stable: 11 Decline: 4	80% had radiological success. 88.57% had no progression. 11.43% collapsed.	Progression = ↑ARCO stage or collapse >2 mm compared to pre-treatment
L. Massari et al. [4]	Prospective	68	Steinberg staging used	-	Primary: 34 Steroids: 17 Trauma: 5	SPT BIOSTIM pulse generator used. Single voltage pulses Frequency: 75 Hz Time: each pulse at 1.3 ms Duration of tx: 8 h/day for 6 months	Core decompression and autologous bone graft (from proximal metaphysis and femoral neck)	X-ray and MRI at 1, 3, 6, 12, 24 months from surgery. After, X-ray yearly and MRI every 2 or 3 yr. Mean follow-up = 5.8 yrs. Steinberg II: 81% no pain and limping, good radiographic results. Steinberg III: 70% success Steinberg IV: 53% good clinical results. 27% good radiographic results.		2 patients needed total hip arthroplasty: - Bilateral in 1 patient (Steinberg III) - Unilateral in 1 patient (Steinberg IV)
L. Massari et al. [2]	Retrospective	66 (76 hips)	Ficat staging used. I: 31 II: 22 III: 23	1/3 were Ficat stage III Intense pain and significant functional restriction	Primary: 51 Secondary (alcohol, trauma, steroids): 15	SPT BIOSTIM pulse generator used: Duration of tx: 8 h/day for 6 months (mean duration = 5+/−2 months)	NSAIDS for pain. Non-weight bearing advised but only 50% complied.	Mean follow-up = 28 months X-ray at follow-up with CT and MRI confirmation if available. Ficat I + II: 50/53 hips preserved; 3 Ficat II had progression Ficat III: 12 hips had progression Ficat I: 45% improved to stage 0; 45% unchanged; 10% worsened to stage II Ficat II: 35% improved to stage I; 50% unchanged; 25% worsened to stage III Ficat III: 0% improved; 50% unchanged; 50% worsened to stage IV	15 of 76 hips progressed. **Pain** - 35 were pain free after 60 days of treatment. - 17 had pain of moderate intensity. - 14 still had intense pain. **Hip joint function** - Normal in 46% - Sufficient in 39% - Insufficient in 15%	The hips that progressed led to severe degenerative OA that required surgery.
C. Windisch et al. [5]	Prospective	35	ARCO staging used. **Group 1** IIA: 3 IIB: 8 IIC: 7 IIIC: 4 **Group 2 (non-PEMF)** IIA: 4 IIB: 9 IIIB: 2 IIIC: 3	Group 1: 3/19 pts. had bilateral involvement Group 2: 2/16 had bilateral involvement	-	Magnetodyn – external magnetic field coil and an invasive bipolar induction screw. Frequency: Sinus shaped external magnetic field of ~20 Hz Magnetic flux density: ~5mT Voltage: ~700 mV induced Electric field strength: 50-700 mV/cm	Curettage, autologous bone grafting (from greater trochanter and proximal femur).	Follow-up checks at 6 and 12 months: clinical exam, clinical evaluation – modified Harris Hip score, Merle D’Aubigne hip score, VAS; imaging – X-ray with pelvic view and axial projection of hip, bilateral MRI. **Group 1:** - 2 stage 2C patients had THA - 2 stage 3C patients had THA **Group 2:** - 1 stage 2B patient had THA - 1 stage 3B patient had THA - 2 stage 3C patients had THA **Comparing clinical outcomes of group 1 and 2:** - No significant difference between groups for D’Aubigne score. - No significant difference in Harris Hip score. - No significant difference in VAS. - No significant improvement/deterioration as a result of the procedure in group 1. - Both procedures promising up to stage 2A.		18% of patients in Group 1 had to have THA. 22% of patients in Group 2 had to have THA.
M.E. Steinberg et al. [11]	Prospective	285 (406 hips)	Steinberg staging used. I: 62 hips II: 133 III: 13 IV: 85 V: 4	-	Primary: 10% Steroids: 38% Alcohol: 37% Alcohol + Steroids: 15% Trauma: 12%	Group 1: Constant DC via cathode wire coiled about the graft and attached to an Osteostem/ Orthofuse Group 2: Capacitative coupling via surface electrodes applied anteriorly and posteriorly to the skin over the femoral head and connected to a portable power unit.	Core decompression and bone grafting	Post-operative evaluation by Harris Hip score, AP + lateral X-rays, taken at 3, 6,12, 18, 24 months and then yearly/two yearly thereafter. Mean follow-up = 46 months **DC group** - Radiographic progression in 70%. - Mean progression: 2/3 stage - Mean 5 point improvement in HHS (64% improved or remained unchanged) - 41% needed THA **Control** - 79% radiographic progression - Mean progress: 1 1/3 a stage - Mean 3 point drop in HHS - 43% improved or unchanged - 37% needed THA **Capacitive coupling** - Clinically and radiographically, 42% improved or remained unchanged - 25% needed THA **Control** - Radiographically and clinically, 50% improved or remained unchanged - 20% needed THA	-	-
M.E. Steinberg et. Al [15]	Prospective + historical control	116 hips 55 hips for historical controls 40 hips for disease progression (separate studies)	Steinberg staging used. Mean stages: Non-stimulated: IIIA Stimulated: IIIB	Non-stimulated: IIIA Stimulated: IIIB *Mean Harris scores* Non-stimulated: 65 Stimulated: 65	-	Constant DC electrical stimulator coiled longitudinally, delivering Intensity: 20microA Duration: 24 h a day for 6 months.	Core decompression and grafting	Mean follow up time = Non-stimulated: 33 months Stimulated: 44 months Electrical stimulation gave better Harris scores, less roentgenographic progression, but similar need for arthroplasty. No fractures or complications	No electricity: IVB Electricity: IVA *Mean Harris scores* **No electricity**: 62 **Electricity**: 70	Nil
Bassett et al. [10]	Prospective	95 (118 hips)	Steinberg staging used. I: 0 II: 12 III: 3 IV: 79 V: 21 VI: 3	*Charnley modification of Merle D’Aubigne-Postel system* Mean scores = Pain:3.9 Function: 3.7 Mobility: 5.2 Total: 12.8	Primary: 44 Trauma: 17 Alcohol: 9 Steroid: 46 Sickle cell: 2	Helmholtz-aiding coils, mounted anteriorly & posteriorly on splint. 7″ diameter coils, 6–8″ apart on brace. Single 12″ diameter contoured coil on femoral head with Velcro around pelvis. Duration: 8–10 h/day, discontinued ″1 year. No changes with weight-bearing	Nil	Mean follow up = 5.3 years; every 2/3 months in 1st year, then 3–6 months after PEMF treatment. Average 4.1 years prior. 9 hips (60%) in Stage II-III improved (3 returned to normal), 90 (76%) hips in stages IIA to VI stayed same, 19 (16%) in IVA to VC worsened (<2 mm collapse). Joint space width increased on average 1 mm in 17 of these 19, but most of these showed clinical improvement	One year: 15 Most recent: 15.8	20 required surgical procedures
R.K. Aaron et al. [6]	Prospective	77 (106 hips)	Ficat staging used. PEMF II: 23 III: 33 Core decompression II: 26 III: 24	Modified D’Aubigne scale: actual scores not given	-	Coil positioned over greater trochanter. Single pulse Frequency: 72 Hz, quasirectangular, 380microsec. Duration: 8 h/day, 12–18 months.	Core decompression in non-PEMF group	Mean follow-up = 3 years *Percentage demonstrating both clinical & roentgenographic success* PEMF: 52% Core: 20%	Clinical success (based on Modified D’Aubigne scale): PEMF: 38 hips (68%) Core decompression: 22 hips (44%)	Nil
Steinberg et al. [14]	Prospective + historical control	40 (40 hips) 55 control hips	Steinberg staging used. Stimulated I: 3 II: 16 III: 1 Non-stimulated I: 4 II: 16	Harris score Stimulated: 94 Unstimulated: 75 Mean stage: Stimulated: IIB Unstimulated: IIA	Steroids: 19 Alcohol: 13 Other: 4 Idiopathic: 4	2 capacitive-coupling units (self-adhering electrodes) over femoral head Frequency: 60 kHz Duration: 24 h Intensity: 5 V peak-peak amplitude.	All 40 patients had core decompression + grafting	*Harris score* Stimulated: 82 Unstimulated: 76 *Clinically unchanged* Stimulated: 42% Unstimulated: 50% *Mean stage* Stimulated: IIIA Unstimulated: IIIA No significant difference *Comparison with conservative* Core: 54% progress, 23% required total hip arthroplasty Conservative: 81%progress, 69% required total hip arthroplasty	-	25% of stimulated & 20% of unstimulated hips required total hip arthroplasty.
R.K. Aaron et al. [2, 6].	Prospective	264 (373 hips)	Steinberg staging	Radiographic progression	-	PEMF	-	Mean follow-up = 35 months Stage I: all hips conserved. 75% radiographic progression Stage II: 77% preserved. 54% radiographic progression Stage III: 53% preserved. 68% radiographic progression	-	-

*AVN* Avascular Necrosis, *ARCO* Association Research Circulation Osseous, *CD* Core Decompression, *PEMF* Pulsed Electromagnetic Field, *DC* Direct Current, *NSAIDs* Non-steroidal Anti-Inflammatory Drugs

### Retrospective studies

In the two retrospective studies, a total of 117 patients or 146 hips with symptomatic, non-collapsed avascular necrosis of the femoral head were included [2, 3]. In both studies, patients were treated with PEMF for 8 h a day for 6 months. Cebrian et al. demonstrated that electromagnetic stimulation in femoral heads of ARCO stages I and II led to a survival percentage of 88.57% of the heads on radiographic assessment [3]. Similarly, the paper by Cadossi et al. revealed that PEMF preserved 90% of the femoral heads of Ficat I, 75% of Ficat II and 50% of Ficat III; there were even improvements in the staging of 45% of Ficat I hips to stage 0 and 35% of Ficat II hips to stage I. Functionally, 46% of patients achieved normal hip function and 39% achieved sufficient hip joint function at the end of treatment. As for pain scores, the study found that 53% of patients were pain free after treatment with PEMF while 26% had pain of moderate intensity [2]. Likewise, Cebrian et al. also noted improvement on the D’Aubigne pain scale in 78.57% of the hips [3]. Nevertheless, in both studies, there were some hips that eventually progressed to collapse. Cebrian’s study had a total of eight femoral head collapses, all of which were of ARCO stage II (*n* = 50) whereas Cadossi found that 15 (three Ficat II and 12 Ficat III initially) of the 76 hips had radiographic progression that led to the development of severe osteoarthritis requiring hip arthroplasty [2, 3].

### Prospective studies

#### PEMF

In six prospective studies, the effect of PEMF therapy as an adjunct to other treatments (core decompression and bone grafting) was evaluated.

In an Italian study conducted by Massari et al., 68 patients with ONFH were treated with core decompression, autologous bone grafts and PEMF. Of those with Steinberg stage II scores, 81% had good results radiographically and clinically had no pain or limp. Similar results were seen in stage III patients but only in 70%. This is further reduced in stage IV patients where only 27% had good radiographic outcomes and 53% had good clinical results. Two patients (one stage III and one stage IV) required total hip arthroplasty (THA) [4].

Windisch et al. divided 35 patients into two groups, one treated with curettage, bone grafting and PEMF (*n* = 19) and one with curettage and bone grafting without PEMF (*n* = 16). In the group that underwent PEMF, four patients in total (18%) had to have THA – two of these patients were ARCO stage II C and the other two were stage III C. On the other hand, the non-PEMF group also had four patients (22%) who required THA. However, one was stage II B, one stage III B and two stage III C. However, interestingly, clinical evaluation of both arms revealed no significant difference in pain and functional scores [5].

In another prospective study, Aaron et al. compared the effectiveness of PEMF against core decompression in Ficat stages II and III hips. Based on clinical response (using a modified D’Aubigne scale), clinical success was determined as marginal pain with retention of the femoral head. They found that 68% of those treated with PEMF were clinically successful, compared to 44% of those treated with core decompression. Roentgenographically, 39% showed progression in those treated with PEMF, versus 64% of those treated with core decompression [6].

Aaron et al. compared patients with stage II and III lesions receiving core decompression and PEMF as adjunct therapy, to core decompression alone. They although there was no difference in joint survival, radiographically, stage II hips showed a significant increase in joint stabilisation with PEMF therapy (77% versus 44% in core decompression alone). Stage III hips receiving PEMF also demonstrated clinical improvement [1, 2, 6].

In two prospective studies, the use of pulsed electromagnetic field therapy in the treatment of osteonecrosis of the femoral head without use of or comparison to additional management (eg: core decompression, bone grafts) was examined [2, 10]. Aaron et al. found that based on need for subsequent joint replacement, the greatest advantage was seen in Steinberg Stage I hips, where none required surgery. 77% of stage II hips were conserved, although there was no statistically significant difference between these and stage I hips. However, of the stage III hips, only 53% were conserved, showing a statistically significant decrease compared to stage II hips. Radiologically, the effect of electrical stimulation was less pronounced. In stage I hips, 75% showed progression, notably more than in stage II and III, where 54% and 68% demonstrated progression [2, 6]. In the other study, Bassett et al. quantified the response to PEMF therapy using the Steinberg staging method. They found that 9 hips showed improvement, and they were all in stages II to III, demonstrating a 60% improvement rate. Of these 9 hips, 3 of these returned to a normal structure. 90 hips across all stages (76%) showed no improvement or deterioration, while 19 hips (16%) showed a deterioration of <2 mm further femoral head collapse [10].

### Direct current stimulation and capacitive coupling

Steinberg et al. conducted several studies comparing the outcome of hips that received electrical stimulation, and those that did not [11, 14, 15]. In one paper, he looked at two groups of patients, one who received direct current (DC) stimulation and one who had capacitive coupling (CC) [11], both in addition to core decompression and grafting. The results of the former group showed radiographic progression in 70% compared to 79% in control hips; there was a mean 5 point improvement in the Harris Hip score whereas the control group had a mean 3 point drop instead; however, 41% of hips treated with DC required THA compared to 37% of control hips. The CC group showed less promising results, with 42% of hips either clinically and radiographically improving or remaining unchanged compared to 50% in the control group; 25% of stimulated hips eventually needed THA versus 20% of unstimulated hips [11].

Steinberg et al. also compared non-operative management with core decompression and grafting alone, and with DC as adjunct. They found that electrical stimulation showed an improvement in number of hips and average extent of roentgenographic progression, albeit not a significant difference. Electrical stimulation also gave better Harris scores, with 64% showing improvement or remained unchanged, versus 43% in the core decompression alone group. Requirement for hip replacement was similar with or without electrical stimulation. Both groups were superior in all aspects compared to non-operative management [15].

In another similar study, Steinberg et al. compared non-operative management, core decompression with grafting, and CC as adjunct to decompression and grafting. They found that no significant difference was found when CC was used, based on roentgenographic progression, clinical evaluation, and hips requiring replacement. However both groups were superior to non-operative management [14].

## Discussion

Osteonecrosis of the femoral head is a debilitating disease which generally occurs in the younger population. Multiple studies have shown that once the roentgenographic changes are established, the disease normally progresses to femoral head collapse requiring joint replacement. Since the group of individuals affected by this condition is usually active, hip replacement in these placements are widely regarded as a last resort as the long term outcomes are less than ideal. Therefore, the general clinical approach to these patients is femoral head preservation and various methods have been sought out. Amongst these methods, core decompression (CD) stands out as a conservative technique that has greater success rates in early disease. The principle behind CD is to lower the intraosseous pressure which has been found to be raised. Theoretically this addresses the relative ischaemia while simultaneously stimulating a vascularized healing response [16]. Two of the Steinberg studies analysed in this review showed that CD demonstrated an improvement in outcome over non-operative treatment [14, 15].

The other method evaluated in the studies is biophysical stimulation (either by PEMF or electrical stimulation via DC or CC). The rationale behind the use of biophysical stimulation is its anti-inflammatory actions which prevent cartilage breakdown and promote angiogenesis, thus limiting the extent of necrosis [2, 4]. Moreover, it encourages bone formation via stimulation of osteoblasts and inhibition of osteoclasts [2, 4], thus slowing the breakdown of structural integrity [6]. In particular, PEMF has been proposed to exert its effects based on the following three concepts: Wolff’s law, the piezoelectric effect and streaming potentials [17].

Wolff’s law states that bones respond to mechanical loads under which they are placed; compression results in osteogenesis on the side compressed and simultaneous resorption on the contralateral side [18]. This occurs via a process called mechanotransduction whereby mechanical signals are transformed into biochemical ones [19].

The piezoelectric effect describes the phenomenon where certain materials demonstrate an ability to generate negative and positive potentials when subjected to mechanical strain. In bone, the piezoelectric nature of hydroxyapatite and collagen results in a negative potential generated during compression and a positive one when the stress is relieved. Notably, the piezoelectric effect is reversible, hence the mechanical stress can be induced with the application of an electric field [20].

In cartilage, streaming potentials refer to the movement of positively charged ions across negatively charged proteoglycans during mechanical stress, generating an electric current which may stimulate chondrocytes [21].

Therefore, a possible mechanism of PEMF application is the induction of a mechanical strain via the converse piezoelectric effect, thus inducing osteogenesis via Wolff’s law, as well as chondrocyte stimulation [17].

### PEMF

In the present review, studies examining the effect of PEMF, whether alone or in combination with other treatments, generally showed some benefit when PEMF was administered. As a treatment used on its own, PEMF was shown to preserve majority of femoral heads (80.2% by Cadossi (2), 88.57% by Cebrian [3], 83.9% by Bassett [10] with these benefits being more pronounced in hips of earlier stages, namely Ficat I and II and Steinberg II and III, and decreasing as severity increased. Remarkably, PEMF has also been shown to reverse the disease progression across 2 of these studies; Bassett et al. found 9 hips demonstrated improvements with 3 of these even returning to normal [10], while Cadossi et al. showed improvements in Ficat stages [2].

Additionally, it was found that PEMF was also effective in improving symptoms of osteonecrosis. Cebrian and Cadossi both found that significant proportions of patients who received PEMF therapy eventually experienced an improvement in pain or even became pain free [2, 3]. Moreover, Massari et al. found that though the efficacy of PEMF decreased overall with increased Steinberg staging, there was greater clinical than radiographic benefit seen in those with Steinberg IV hips [4], further reinforcing the potential of PEMF to alleviate pain in these patients. Conversely, Windisch et al. showed that there was no difference in clinical outcomes between patients who received PEMF and those who didn’t. However, unlike the other papers, the method of inducing the electromagnetic field in this study was an invasive one via a bipolar induction screw through the femoral head [5]. This may have contributed to the discrepancy, as discussed later in the section on DC therapy.

A notable limitation to these studies is the lack of comparison to pain outcomes in non-operative management, hence making it difficult to ascertain the actual degree of improvement. However, Aaron et al. found that more patients who received PEMF alone experienced less pain than patients who received core decompression alone [6]. This is significant as it is the only study that directly compared outcomes of PEMF therapy to the current most widely-accepted conservative treatment method, and it showed a clear advantage of PEMF over core decompression.

These findings show that PEMF therapy is a promising technique, especially for the management of early stage disease.

### Direct current stimulation

Two studies examined the effect of electrical stimulation as an adjunct to core decompression and grafting with varying results. One study showed improvements in Harris Hip scores and less roentgenographic progression in electrically stimulated hips via DC, although the percentage of patients needing THA in both groups was the same [15]. Similarly, in another study, femoral heads that were treated with DC had better radiological and clinical outcomes than the control group, with an average progression of two thirds a stage compared to one and a third a stage respectively [11]. However, surprisingly, more hips from the DC group eventually required THA (41% vs 37%) [11]. Although there appears to be a small benefit with DC stimulation, its efficacy should be considered in the context of it being an invasive procedure. Due to the study designs, DC application was only evaluated as an adjunct therapy to core decompression and grafting, where it showed no extra benefit. Therefore, more research is required to assess its efficacy as a technique alone. Yet, randomised double-blind controlled trials may not be suitable in this instance, as the control group would likely have to receive insertion of a placebo device, which is ethically problematic [22]. As such, future trials should compare DC therapy alone to other techniques alone, or vary the protocols used in terms of voltage and length of stimulation.

### Capacitive coupling

Patients who received CC fared worse than those who did not. In one study, hips that were stimulated showed poorer outcomes in all parameters: roentgenographic progression, HHS and Steinberg staging [14]. The other study also revealed comparable findings with unstimulated hips faring better [11]. This is noteworthy because CC is another non-invasive method of applying electric fields, yet the results yielded are significantly worse than those of PEMF. One interesting difference we identified between the PEMF and CC groups is the duration of stimulation: all the patients who were treated with PEMF had it administered 8 h a day whereas those who received CC in one of the studies had their affected hips stimulated nearly 24 h a day [14]. This may explain the large discrepancy in results between the two modalities; it may be that CC would have similar effects to PEMF if the protocols used were more similar. This difference also makes it difficult to directly compare these studies, hence, more controlled clinical trials are needed before any concrete conclusion can be made about the effectiveness of CC compared to PEMF, with emphasis on evaluating the optimal protocol for CC application.

### Prognostic factors

In the paper by Cebrian et al., it was noted that in addition to the presence of certain radiological features, having a femoral head with a greater than 15% necrotic area influenced the likelihood of progression as well. Moreover, they identified that all of the femoral heads that went on to have roentgenographic progression had predominantly lateral involvement [3]. Similarly, Steinberg et al. noted that hips with small lesions fared significantly better than those with intermediate and large lesions [11]. As such, lesion size and its location may be important prognostic markers, and are parameters that haven’t been addressed in other papers.

Finally, another important point to note is that many of these papers either did not take into consideration the aetiologies of the disease, or did not evaluate the outcomes according to aetiologies. With regards to PEMF therapy, Bassett et al. noted that corticosteroid use as an aetiology may have influenced response [10], while Cadossi et al. proposed that idiopathic lesions may be more sensitive [2]. Steinberg et al. mentioned that patients who have had alcohol and steroid use as disease aetiologies may have had poorer outcomes, although the difference was not statistically significant [11]. Causes of the disease may be a confounding factor; secondary lesions may be less responsive to treatment due to their ongoing nature, for example steroid use for treatment of another disease should not be interrupted [4]. Studies to date on this topic are limited in number, size, and quality of research methodology. There is heterogeneity in the methodology used (eg: dosage of electrical stimulation and follow up period) so a meta-analysis would not yield any meaningful data on the outcomes of interest. Hence this article shows the best available evidence on electrical stimulation in the management of AVN in the femoral head. Therefore, it is recommended that future research investigate the relationship between treatment outcomes and disease aetiologies.

## Conclusion

The outcomes of stimulated femoral heads with osteonecrosis with PEMF have been encouraging, with the improvement in both radiographic and clinical parameters, especially in early Ficat stages. Given its non-invasive nature and potential to stop or reverse the disease process, PEMF is an especially promising area of research. However, the technique is perhaps hindered by the fact that its application is generally cumbersome and requires significant compliance on the part of the patients; the devices often require long hours of use for many months (e.g. 8 h a day for 6 months (2, 3, 4, 6, 10), and precise placement of the coils, typically requiring splints [3, 4, 6, 10]. On the other hand, other techniques of electrical stimulation such as with DC or CC have shown equivocal results. In essence, more trials need to be completed to ascertain the indications for and complications of the use of electrical stimulation in avascular necrosis of femoral heads, and thus derive an optimal protocol.

## Abbreviations

ARCO: Association Research Circulation Osseous
AVN: Avascular necrosis
CD: Core Decompression
CT: Computed tomogram
DC: Direct current
NSAIDs: Non-steroidal Anti-Inflammatory Drugs
PEMF: Pulsed Electromagnetic Field

## References

[1] Aaron RK (1994). Treatment of osteonecrosis of the femoral head with electrical stimulation. Instr Course Lect.

[2] Massari L, Fini M, Cadossi R, Setti S, Traina GC (2006). Biophysical stimulation with pulsed electromagnetic fields in osteonecrosis of the femoral head. J Bone Joint Surg Am.

[3] Cebrian JL, Milano GL, Francis A, Lopiz Y, Marco F, Lopez-Duran L. Role of electromagnetic stimulation in the treatment of osteonecrosis of the femoral head in early stages. J Biomed Sci Eng. 2014:252–7.

[4] Massari L, Fini M, Cadossi R, Setti S, Traina GC (2009). Biophysical stimulation in osteonecrosis of the femoral head. Indian J Orthop.

[5] Windisch C, Kolb W, Rohner E, Wagner M, Roth A, Matziolis G (2014). Invasive electromagnetic field treatment in osteonecrosis of the femoral head: a prospective cohort study. Open Orthop J.

[6] Aaron RK, Lennox D, Bunce GE, Ebert T (1989). The conservative treatment of osteonecrosis of the femoral head. A comparison of core decompression and pulsing electromagnetic fields Clin Orthop Relat Res.

[7] Duffy, G. P.; Berry, D. J.; Rowland, C.; Cabanela, M. E. Primary uncemented total hip arthroplasty in patients <40 years old: 10- to 14-year results using first-generation proximally porous-coated implants J Arthroplasty 2001;16(8 Suppl 1):140–4. 10.1054/arth.2001.28716 11742466

[8] Crowther, J. D.; Lachiewicz, P. F. Survival and polyethylene wear of porous-coated acetabular components in patients less than fifty years old: results at nine to fourteen years. J Bone Joint Surg Am 2002;84-a(5):729–35. 10.2106/00004623-200205000-00005 12004013

[9] Luben RA (1982). Effects of electromagnetic stimuli on bone and bone cells in vitro: inhibition of responses to parathyroid hormone by low-energy low-frequency fields. Proc Natl Acad Sci.

[10] Bassett CA, Schink-Ascani M, Lewis SM (1989). Effects of pulsed electromagnetic fields on Steinberg ratings of femoral head osteonecrosis. Clin Orthop Relat Res.

[11] Steinberg ME, Larcom P, Strafford B, Hosick WB, Corces A, Bands RE (1997). Treatment of osteonecrosis of the femoral head by Core decompression, bone grafting, and electrical stimulation. UPOJ.

[12] Moher D, Liberati A, Tetzlaff J (2009). Preferred reporting items for systematic reviews and meta-analyses: the PRISMA statement. Ann Intern Med.

[13] von Elm E, Altman DG, Egger M (2007). The strengthening the reporting of observational studies in epidemiology (STROBE) statement: guidelines for reporting observational studies. Prev Med.

[14] Steinberg ME, Brighton CT, Bands RE, Hartman KM (1990). Capacitive coupling as an adjunctive treatment for avascular necrosis. Clin Orthop Relat Res.

[15] Steinberg ME, Brighton CT, Corces A, Hayken GD, Steinberg DR, Strafford B (1989). Osteonecrosis of the femoral head. Results of core decompression and grafting with and without electrical stimulation Clin Orthop Relat Res.

[16] Gupta AK, Frank RM, Harris JD, McCormick F, Mather RC, Nho SJ (2014). Arthroscopic-assisted Core decompression for osteonecrosis of the femoral head. Arthroscopy Techniques.

[17] Trock DH. ELECTROMAGNETIC FIELDS AND MAGNETS: Investigational treatment for musculoskeletal disorders. Rheum Dis Clin N Am 2000;26(1):51–62. 10.1016/s0889-857x(05)70119-8 10680193

[18] Frost HM (2004). A 2003 update of bone physiology and Wolff's law for clinicians. Angle Orthod.

[19] Huang C, Ogawa R (2010). Mechanotransduction in bone repair and regeneration. FASEB J.

[20] Fukada E, Yasuda I. On the Piezoelectric Effect of Bone. ​J Phys Soc Jpn. 1957;12:1158–62. http://dx.doiorg/101143/JPSJ121158.

[21] Kim YJ, Bonassar LJ, Grodzinsky AJ (1995). The role of cartilage streaming potential, fluid flow and pressure in the stimulation of chondrocyte biosynthesis during dynamic compression. J Biomech.

[22] Griffin M, Bayat A. Electrical stimulation in bone healing: critical analysis by evaluating levels of evidence. Eplasty. 2011;11 PMC3145421 21847434

